# The Impact of School Burnout on Life Satisfaction Among University Students: The Mediating Effects of Loneliness and Fear of Alienation

**DOI:** 10.3390/bs15081083

**Published:** 2025-08-09

**Authors:** Taeeun Shim, Eunsun Go

**Affiliations:** 1 Department of Education, College of Education, Dongguk University, Seoul 04620, Republic of Korea; shim2593@dongguk.edu; 2 National Institute for International Education, Seongnam 13557, Republic of Korea

**Keywords:** school burnout, life satisfaction, loneliness, fear of alienation, university students, structural equation modeling

## Abstract

University students face increased stress and potential school burnout amid rapid digital transformation and competitive academic environments, yet little is known about how socioemotional processes explain the link between burnout and life satisfaction. This study examined how school burnout affects life satisfaction, mediated by loneliness and fear of alienation. A cross-sectional survey of 1783 students was conducted to measure school burnout, loneliness, fear of alienation, and life satisfaction. Structural equation modeling showed that school burnout had a significant negative direct effect on life satisfaction, mediated by loneliness. Higher burnout predicted greater loneliness, which in turn lowered life satisfaction. Although school burnout positively predicted fear of alienation, fear of alienation showed a complex association, with a positive direct path to life satisfaction. However, when loneliness was considered in the full mediation model, the overall indirect effect remained significantly negative. The sequential mediation pathway (school burnout → loneliness → fear of alienation → life satisfaction) highlighted how students’ social disconnection can intensify concerns about exclusion, ultimately affecting their well-being. These findings extend the literature by clarifying the socioemotional mechanisms linking school burnout and life satisfaction. Interventions should address academic demands and bolster emotional support, including resilience training, social skills development, and community-building programs, to mitigate loneliness and manage alienation concerns, thereby promoting students’ life satisfaction and psychological wellness.

## 1. Introduction

University students face various challenges related to the rapid changes in digital environments and complex social demands (Koivuneva & Ruokamo, 2022). Upon entering university, rather than immersing themselves in the core pursuits of academia and campus life, students experience psychological stress from practical demands, such as managing grades, working part-time, obtaining certifications, preparing for employment, and navigating interpersonal relationships (Kum, 2018; Reddy et al., 2018). In particular, academic stress and anxiety about future uncertainty may negatively affect students’ psychological well-being and overall life satisfaction (J. H. Park & Kim, 2017; Ribeiro et al., 2018).

In this context, school burnout among university students has emerged as an important research topic, both theoretically and practically. School burnout encompasses chronic fatigue, feelings of helplessness toward academic tasks, and emotional exhaustion experienced during the process of studying (M. R. Lee & Park, 2018; Vansoeterstede et al., 2023). If this phenomenon persists, it can not only cause academic achievement to decrease but can also increase the likelihood of subsequent anxiety or depressive symptoms, thereby reducing life satisfaction (May et al., 2014, 2015). Therefore, an in-depth understanding of the underlying mechanisms and effective prevention and mitigation strategies for school burnout are urgently needed.

In this context, university students, as future professionals, undergo the critical transition of emerging adulthood, during which life satisfaction is directly linked to vocational and social adjustment (Arnett, 2016). School burnout can be a key factor undermining their motivation for adult life. If not effectively managed, school burnout is likely to transition into job burnout after students graduate and enter the professional field (Maslach, 2018).

Stress related to adapting to university life and entering society negatively affects students’ psychological stability and life satisfaction. In particular, loneliness and fear of alienation have been identified as key factors that intensify feelings of isolation and psychological stress in a digital society (Przybylski et al., 2013). Loneliness reflects a state of emotional depletion resulting from disrupted social connections, whereas fear of alienation denotes anxiety regarding the possibility of future exclusion. These two factors operate as distinct mechanisms that influence school burnout and life satisfaction (Kaufmann & Vallade, 2022). Moreover, prior research has indicated that these psychological states function differently depending on individual resilience and social support systems and have important mediating effects on overall well-being (Conlin et al., 2016; Rubin, 2017).

Nevertheless, the effects of loneliness and fear of alienation on the relationship between school burnout and life satisfaction have not yet been empirically clarified. Therefore, this study used loneliness and fear of alienation as mediating variables to propose a more refined structural model to explain the effects of school burnout on life satisfaction.

## 2. Theoretical Background and Hypotheses

### 2.1. School Burnout

Burnout encompasses emotional exhaustion, cynical attitudes, and reduced efficacy resulting from prolonged stress. Although primarily studied in occupational contexts, research on burnout in academic settings has recently increased (Hakanen et al., 2008). Among university students, school burnout refers to a state of psychological exhaustion arising from a chronic imbalance between academic demands and personal resources and is characterized by emotional exhaustion, cynicism, and reduced efficacy (M. R. Lee & Park, 2018; Maslach et al., 2001; Salmela-Aro et al., 2009; Schaufeli et al., 2002). Reduced self-efficacy undermines learning motivation and deepens emotional exhaustion and helplessness, thus becoming a key driver of students’ failure to achieve their academic and career goals (Bask & Salmela-Aro, 2013).

School burnout among university students is attributable to both individual and social factors. Individual factors stem from an imbalance between excessive academic demands (e.g., heavy workload and exam pressure) and insufficient personal resources (e.g., self-efficacy and social support). Social factors include the increase in online classes after the COVID-19 pandemic and rapid changes in digital environments (Koivuneva & Ruokamo, 2022). In particular, disruptions in social relationships and reduced interactions in digital settings can intensify students’ feelings of psychological isolation and burnout, which may contribute to academic dropout in the short term and increase the risk of burnout after entering the professional workforce in the long term (Jo, 2020). Students with high levels of school burnout are more likely than their peers to experience emotional problems, such as depression, anxiety, and self-deprecation, as well as interpersonal conflicts and social isolation, potentially resulting in decreased life satisfaction and a broader maladjustment to university life (J. H. Kim et al., 2013; M. R. Lee & Park, 2018; Shin & Yang, 2016). Thus, school burnout among university students is an important research topic that not only relates to declining academic performance but also has serious implications for future career attainment and social adaptation (Schaufeli et al., 2002; Wang et al., 2022).

### 2.2. Life Satisfaction

Life satisfaction is a core component of subjective psychological well-being, reflecting the extent to which individuals positively evaluate and accept their lives (Neugarten et al., 1961). For university students, life satisfaction is linked to academic achievement, career decision-making, and long-term quality of life, making it important beyond happiness alone. Prior research has shown that factors such as self-esteem, career decision self-efficacy, grit, and school adjustment are positively associated with life satisfaction among university students (Kang & Yang, 2019; Shin et al., 2015). These factors are reportedly crucial for successful university experiences and subsequent career attainment. Conversely, psychological problems such as social withdrawal, aggression, depression, anxiety, and stress can negatively predict life satisfaction (Kumar et al., 2016). For university students, school burnout exerts a strong negative influence on life satisfaction, which can be attenuated depending on individual psychological resilience and social support levels (Shankland et al., 2019; Wang et al., 2022). Recent life satisfaction research has been grounded in the subjective well-being theory, which conceptualizes life satisfaction as a combination of emotional experiences and cognitive evaluations (Diener et al., 2003). Specifically, the subjective well-being theory posits that life satisfaction comprehensively reflects positive affect, negative affect, and cognitive appraisal of one’s life, determined by the interactions between an individual’s emotional state and environmental circumstances (Pavot & Diener, 2008). Therefore, this study examines the impact of school burnout on life satisfaction among university students, as well as the roles that loneliness and fear of alienation play in this relationship.

### 2.3. Loneliness and Fear of Alienation

Loneliness reflects a subjective emotional and social deficiency experienced in one’s social relationships and is known to have direct negative effects on psychological well-being and life satisfaction (Cacioppo et al., 2006). In modern society, loneliness is one of the fastest growing factors affecting psychosocial health (Rubin, 2017). University students frequently experience loneliness as they transition from living with their family to being independent and forming new social networks, which can impair academic performance and social adjustment, thereby increasing school burnout and decreasing life satisfaction (K. T. Lee et al., 2013).

Fear of alienation is a psychological state involving anxiety and concerns about being excluded or alienated in social relationships (Jang & Kim, 2022). Recent studies have indicated associations between fear of alienation and behavioral issues, such as excessive social media use, increased depressive symptoms, and impulsive purchasing. This fear is a major factor that exacerbates feelings of alienation and anxiety among university students in contexts of intense competition and frequent social comparison in digital environments (Yi & Kim, 2018). However, fear of alienation is also strongly linked to interpersonal connections and can sometimes motivate individuals to spend more time in groups rather than solely pursuing personal desires (Conlin et al., 2016). According to the social comparison theory, when personal needs are frustrated, negative emotions arise. In this context, fear of alienation is negatively correlated with life satisfaction and positively correlated with negative emotions (Joo et al., 2018).

Loneliness depletes students’ emotional energy, thus deepening school burnout (Kaufmann & Vallade, 2022). Furthermore, fear of alienation consumes the energy needed to form social relationships, resulting in lower life satisfaction (Przybylski et al., 2013). This study provides an in-depth analysis of the mediating effects of the psychological mechanisms of loneliness and fear of alienation, aiming to comprehensively elucidate the structural relationship between school burnout and life satisfaction among university students.

### 2.4. Hypotheses

This study aims to conduct an in-depth analysis of the relationship between school burnout and life satisfaction among university students and examine the multiple mediating effects of loneliness and fear of alienation in this relationship. Accordingly, the following hypotheses were formulated.

**Figure 1:**
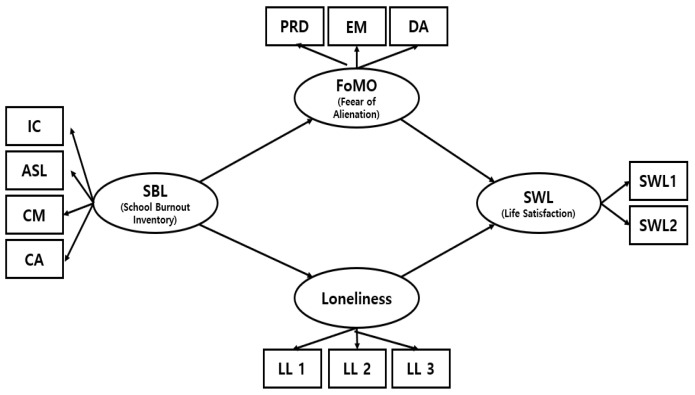
The research model.

The research model that illustrates the hypothesized relationships is presented in Figure 1.

**H1.**  *School burnout negatively affects life satisfaction among university students.*

**H2.**  *School burnout positively predicts loneliness, which in turn negatively predicts life satisfaction*.

**H3.**  *School burnout positively predicts fear of alienation, which in turn negatively predicts life satisfaction.*

**H4.**  *Loneliness and fear of alienation operate in sequence, such that school burnout increases loneliness, which subsequently heightens fear of alienation, ultimately decreasing life satisfaction.*

This study has the following academic and practical implications:

1. By empirically testing the mediating effects of loneliness and fear of alienation on the relationship between school burnout and life satisfaction, this study extends the theoretical horizon of mental health research among university students. Furthermore, it moves beyond simple associations to clarify the underlying mechanisms.
2. Through a multiple mediation analysis using structural equation modeling (SEM), this study elucidates the causal links among the variables more precisely. This goes beyond conventional correlational analyses to propose a more sophisticated theoretical model of the impact of school burnout on life satisfaction through psychological mediators.
3. Conducting an empirical study in domestic (Korean) university students provides context-specific findings, thus helping to concretize knowledge in a field that often relies on international research. These localized insights can inform interventions and policies tailored to the context of Korean higher education.

## 3. Method

### 3.1. Participants

University students were selected as the study population to analyze the effects of school burnout on life satisfaction. The study was approved by the Institutional Review Board of the university. A total of 1783 students participated in the survey. Of these students, 921 (51.7%) were enrolled in humanities, social sciences, arts, and physical education programs, and 862 (48.3%) were enrolled in natural sciences and engineering programs. The sample included 981 (55.0%) male and 802 (45.0%) female participants.

### 3.2. Measures

#### 3.2.1. School Burnout Inventory (SBL)

School burnout was assessed using a scale developed based on Schaufeli et al. (2002). The inventory comprises 20 items rated on a 5-point Likert scale, with higher scores indicating greater levels of school burnout. The subscales are as follows: identity confusion (IC: 6 items), apathy toward school life (ASL: 4 items), classroom maladaptation (CM: 4 items), and career anxiety (CA: 6 items). The identity confusion subscale included items such as *“I don’t know why I am attending school”*. The apathy toward school life subscale included items such as *“I want to give up when I have to do assignments that do not help my career”*. The classroom maladaptation subscale included items such as *“I often feel like lying down during class”*. Finally, the career anxiety subscale included items such as *“I feel anxious because I am not confident about getting a job”*. The internal consistency reliability for the overall scale was high (Cronbach’s α = 0.906).

#### 3.2.2. Loneliness

Loneliness was measured using the UCLA Loneliness Scale developed by Russell et al. (1980). The scale comprises 20 items rated on a 5-point Likert scale, with higher scores indicating greater levels of loneliness. The scale was divided into three subdimensions: emotional isolation (LL1; 7 items), social isolation (LL2; 7 items), and lack of belonging (LL3; 6 items). The emotional isolation subdimension included items such as “*I feel isolated from others*”. The social isolation subdimension included items such as “*I have no one to turn to*”. Finally, the lack of belonging subdimension included items such as “*I feel abandoned*”. In this study, the internal consistency reliability was high (Cronbach’s α = 0.923).

#### 3.2.3. Fear of Alienation (FoMO)

The Fear of Missing Out (FoMO) scale developed by Przybylski et al. (2013) was used to assess fear of alienation in the context of digital and social environments. This instrument comprises 8 items rated on a 5-point Likert scale, with higher scores indicating greater levels of FoMO (interpreted here as fear of alienation). The scale includes three subdimensions: perceived relative deprivation (PRD; 2 items), extrinsic motivation (EM; 4 items), and desire for affiliation (DA; 2 items). The perceived relative deprivation subdimension included items such as *“I am afraid that my friends might be having better experiences than I am”*. The extrinsic motivation subdimension included items such as *“I often worry that I am spending too much time trying to pay attention to others”*. Finally, the desire for affiliation subdimension included items such as *“I feel anxious if I have to miss a scheduled group gathering”*. This study used the full set of 8 items without modification, as recommended by the scale developers. The internal consistency reliability was acceptable (Cronbach’s α = 0.866).

#### 3.2.4. Life Satisfaction (SWL)

Life satisfaction was assessed using the Satisfaction with Life (SWL) scale developed by Diener et al. (1985). The scale comprises 5 items rated on a 5-point Likert scale, with higher scores indicating greater life satisfaction. The scale was divided into two subdimensions: present life evaluation (SWL1; 3 items) and retrospective life evaluation (SWL2; 2 items). The present life evaluation subdimension included items such as *“I am satisfied with my life”*. The retrospective life evaluation subdimension included items such as *“If I could live my life over, I would not change anything”*. In this study, the internal consistency reliability was acceptable (Cronbach’s α = 0.808).

### 3.3. Data Analysis

Data were analyzed using SPSS Statistics version 25 (IBM Corp., Armonk, NY, USA) and AMOS version 20 (IBM Corp., Armonk, NY, USA) to examine the mediating pathways from school burnout to life satisfaction through loneliness and fear of alienation among university students. The specific analysis methods were as follows:

**Step 1: Descriptive and Reliability Analysis**
  - Frequency analyses were conducted to describe the demographic characteristics of the sample.
  - Cronbach’s α coefficients were calculated to assess the internal consistency of each scale.
**Step 2: Correlation and Descriptive Statistics**
  - Pearson’s product–moment correlation analysis was performed to examine the relationships among the study variables.
  - Descriptive statistics (means and standard deviations) were calculated for all key variables.
**Step 3: Structural Equation Modeling for Mediation**
  - Following Anderson and Gerbing’s (1988) two-step approach, SEM was used to examine the mediating effects of loneliness and fear of alienation on the relationship between school burnout and life satisfaction.
  - The measurement models were specified before testing the structural model. For single-factor constructs (loneliness and life satisfaction), item parceling was applied based on factor analysis to form parcel indicators (Kline, 2015).
  - Step 1—Confirmatory factor analysis (CFA): The reliability and validity of the measurement model were evaluated. Model fit was assessed using indices such as χ^2^, χ^2^/*df*, normed fit index (NFI), the Tucker–Lewis index (TLI), the comparative fit index (CFI), and the root mean square error of approximation (RMSEA). Factor loadings, critical ratios (C.R.s), the average variance extracted (AVE), and the composite reliability (CR) were calculated to examine the construct reliability, convergent validity, and discriminant validity.
  - Step 2—Structural model testing: The structural model was estimated using maximum likelihood estimation (MLE). Model fit indices (χ^2^, χ^2^/*df*, NFI, TLI, CFI, RMSEA) were used to assess the overall fit. Path coefficients were tested for statistical significance at the α = 0.05 level. The Sobel test was conducted for mediation pathways to examine the significance of the total and indirect effects.

### 3.4. Measurement Model Assessment

The analysis of the research model followed a two-step approach. First, the fully mediated model was examined. Second, the partial mediation model was estimated (Hoyle & Smith, 1994). To evaluate the model fit while accounting for sensitivity to the sample size, overall fit, parsimony, and interpretability, the following indices were used: χ^2^, RMSEA, NFI, TLI, and CFI (G. S. Kim, 2010; Kline, 2015).

Validating the measurement model is advantageous for identifying potential sources of misfit before testing the structural model (Kline, 2015). CFA was conducted using maximum likelihood estimation (MLE) to assess the fit between the latent constructs and their observed indicators. Model fit was evaluated using the following indices:

- χ^2^ (chi-square), reflecting the degree to which the covariance matrix reproduces the observed data, and
- TLI, CFI, and RMSEA, indicating how well the specified measurement model fits relative to a null model and whether the fit meets the conventional thresholds.

The CFA results for the measurement model indicated an acceptable fit (Kline, 2015): χ^2^ = 709.146 (*df* = 48, *p* < 0.001), TLI = 0.922 (≥0.90), CFI = 0.943 (≥0.90), and RMSEA = 0.088 (≤0.10). Factor loadings (standardized regression weights) for the observed indicators ranged from 0.576 to 0.983 (Table 1), demonstrating satisfactory indicator loadings on their respective latent constructs.

Average variance extracted (AVE) and construct reliability (CR) were examined to assess the reliability and validity of the measurement model. AVE values ranged from 0.509 to 0.598 (threshold ≥ 0.50), and construct reliability values ranged from 0.802 to 1.454 (threshold ≥ 0.70). All exceeded the recommended criteria for convergent validity and internal consistency (G. S. Kim, 2010).

## 4. Results

### 4.1. Descriptive Statistics and Correlation Analysis

Before performing SEM, preliminary analyses were conducted to assess normality and multicollinearity. As Table 2 shows, an examination of multivariate normality indicated that the absolute skewness values for all variables were below 3 and the absolute kurtosis values were below 10, suggesting that the normality assumption was satisfied (Kline, 2015). Furthermore, an assessment revealed no evidence of multicollinearity issues in the present dataset (G. S. Kim, 2010). Correlation analyses for the primary variables showed positive associations between school burnout, fear of alienation, and loneliness (*r* ranged from 0.439 to 0.527), whereas life satisfaction was negatively correlated with school burnout, fear of alienation, and loneliness (*r* ranged from −0.271 to −0.491). Notably, the highest correlation was observed between school burnout and fear of alienation (r = 0.527), whereas the lowest correlation was between life satisfaction and loneliness (*r* = −0.491).

### 4.2. Model Fit and Mediating Effects

**Figure 2:**
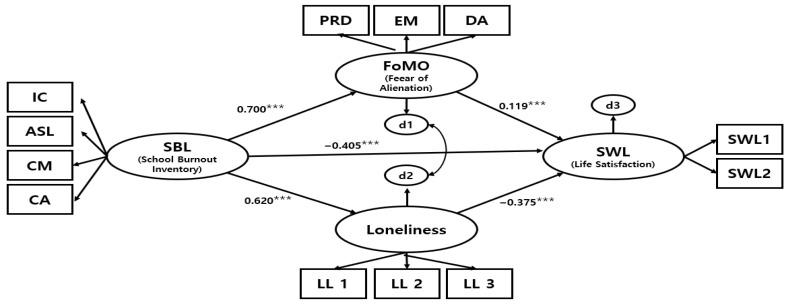
The Structural equation model with standardized path coefficients illustrating direct and indirect effects of school burnout on life satisfaction via loneliness and fear of alienation. *** *p* < 0.001.

As Table 3 shows, the fully mediated model yielded χ^2^ = 492.465 (*df* = 47, *p* < 0.001), NFI = 0.958, TLI = 0.946, CFI = 0.962, and RMSEA = 0.073. The partial mediation model yielded χ^2^ = 416.346 *(df* = 46, *p* < 0.001), NFI = 0.965, TLI = 0.955, CFI = 0.968, and RMSEA = 0.067. These values exceed the conventional thresholds for an acceptable model fit (Kline, 2015). A chi-square difference test indicated that the partial mediation model was a significant improvement over the fully mediated model (Δχ^2^ = 76.119, Δ*df* = 1, *p* < 0.001). The fit indices further showed that the partial mediation model provided a better fit than the fully mediated model. Therefore, the partial mediation model, in which university students’ school burnout affects life satisfaction both directly and indirectly via fear of alienation and loneliness, was selected as the final model. These findings are visually summarized in the final structural equation model with standardized path coefficients (Figure 2).

Table 4 presents the results of examining the path coefficients, which indicate that school burnout, loneliness, and fear of alienation each have significant direct effects on life satisfaction. School burnout directly and significantly predicted loneliness (β = 0.620, *p* < 0.001), fear of alienation (β = 0.700, *p* < 0.001), and life satisfaction (β = −0.405, *p* < 0.001). Loneliness directly and significantly predicted life satisfaction (β = −0.375, *p* < 0.001), and fear of alienation directly and significantly predicted life satisfaction (β = 0.119, *p* < 0.001). Thus, school burnout appeared to increase both loneliness and fear of alienation, while also directly lowering life satisfaction. Life satisfaction was negatively affected by loneliness but positively affected by fear of alienation, indicating a complex pattern that should be interpreted carefully considering the theory and data.

The final structural equation model with standardized path coefficients is shown in Figure 2.

Table 5 presents the direct, indirect, and total effects of school burnout on life satisfaction through loneliness and fear of alienation. As shown in the direct effects column, school burnout exerted a significant negative direct effect on life satisfaction (β = −0.405, *p* < 0.001). The indirect effects column shows that school burnout also had a significant negative indirect effect on life satisfaction via loneliness and fear of alienation (β = −0.149, *p* < 0.001). Consequently, the total effects column, which sums the direct and indirect effects, indicates that the overall effect of school burnout on life satisfaction was significantly negative (β = −0.554, *p* < 0.001).

The significance of these mediating effects was further confirmed using Sobel tests (Table 6). Specifically, the Sobel test showed that the mediating pathway through loneliness was significant (Z = −10.048, *p* < 0.001), as was the pathway through fear of alienation (Z = 4.489, *p* < 0.001). These findings indicate that school burnout decreases life satisfaction both directly and indirectly through increased loneliness and fear of alienation. These results indicate that both mediators contributed significantly to the total effect (MacKinnon et al., 2002).

## 5. Discussion

This study aimed to inform efforts to prevent school burnout among university students by analyzing its effects on life satisfaction, focusing on the direct, indirect, and total effects, with loneliness and fear of alienation as mediators.

First, consistent with previous findings (Wang et al., 2022), students experiencing identity confusion, apathy toward school life, classroom maladaptation, or career anxiety were found to be at greater risk of school burnout, which, in turn, lowered their life satisfaction. This aligns with Wang and Wu (2022) and Mehdinezhad (2015), who showed that maladaptive perfectionism reduces life satisfaction via academic burnout. Moreover, treating study as work (Ouweneel et al., 2011) and insufficient learning resources relative to academic demands have been linked to burnout and are indirectly associated with reduced life satisfaction (Lesener et al., 2020; Scheepers et al., 2024). Hence, the long-term management of school burnout is essential to promote effective stress management and foster a healthy academic environment (Stoliker & Lafreniere, 2015). These results support the subjective well-being theory, which conceptualizes life satisfaction as the integration of emotional experiences and cognitive evaluations (Diener et al., 2003), indicating that negative emotions arising from burnout can adversely affect overall life appraisal.

Second, school burnout indirectly influenced life satisfaction through increased loneliness and fear of alienation. Thus, consistent with prior research, students with higher levels of school burnout experienced greater loneliness, which further diminished their life satisfaction. Loneliness and fear of alienation may be exacerbated in modern digital and competitive contexts, which could accelerate the psychological decline associated with school burnout. This underscores that internalized social emotions, such as loneliness and fear of alienation, are deeply intertwined with students’ psychological well-being (Cacioppo et al., 2006; Kaufmann & Vallade, 2022). Furthermore, according to the study demands–resources model (Lesener et al., 2020), when academic demands (e.g., parental pressure, grades, and evaluations) exceed the available resources (e.g., parental support, effort, and rewards), emotional exhaustion arises, leading to heightened social emotions, such as loneliness.

Third, we demonstrated a sequential multiple mediation effect, wherein school burnout sequentially triggers loneliness and then fear of alienation, thus further decreasing life satisfaction. However, in this study, fear of alienation exerted a positive effect on life satisfaction. This finding suggests that, rather than only being a negative emotion, fear of alienation may also be a social motivational factor that stimulates efforts to restore social connections or satisfy the need for group belonging (Conlin et al., 2016). Thus, it can be interpreted as a source of extrinsic behavioral motivation (Joo et al., 2018).

Emotional support programs that alleviate loneliness and feelings of alienation, in addition to interventions that reduce academic burden, are urgently needed to prevent school burnout and enhance life satisfaction. For example, programs such as resilience-enhancing emotion coaching, social skills training, and group counseling may be effective and should be implemented in coordination with university counseling centers (H. G. Park, 2018; Seo & Kim, 2017). Teaching concrete skills for forming and maintaining close interpersonal relationships and helping students build support networks with their family, peers, and seniors could also be useful in improving students’ life satisfaction (D. H. Lee & Lee, 2021). Furthermore, at the institutional level, universities should establish integrated student support systems that include wellness assessment platforms, the activation of small-group communities, and enhanced access to academic resources.

This study’s findings have substantial practical implications, as university students’ psychological wellness has a long-term impact on successful career entry and job adaptation (Arnett, 2016). Lesener et al. (2020) applied the study demands–resources model to empirically demonstrate that balancing academic demands and resources can alleviate student burnout and enhance life satisfaction, underscoring the need for preventive academic environmental design across the education system and systematic, organized support programs at the institutional level.

## 6. Conclusions and Limitation

This study empirically demonstrated that school burnout negatively affects life satisfaction among university students and that loneliness and fear of alienation significantly mediate this relationship. Notably, loneliness and fear of alienation can readily emerge in digitalized and competitive educational contexts and warrant attention owing to their strong link to decreased quality of life. From the perspectives of the subjective well-being theory and the study demands–resources model, this study’s findings elucidate the complex mechanisms underlying the effects of psychological stress and social isolation on life satisfaction. The results also offer practical implications, including in the development of emotional support systems, community-building programs, and academic support services aimed at enhancing students’ emotional stability and sense of belonging.

However, this study had some limitations. First, the cross-sectional design hinders definitive conclusions regarding the causal relationships between school burnout, loneliness, fear of alienation, and life satisfaction. Thus, future longitudinal research is required to establish temporal causality. Second, because the sample comprised students from a single university, generalizing the findings to other student populations or age groups should be performed with caution. Additionally, the study did not collect detailed information about the age distribution of the participants, which may limit the ability to assess potential age-related differences. Future studies should include diverse institutions, regions, and fields to assess the robustness and generalizability of these results. Third, all variables were measured using self-reported questionnaires, which may introduce common method bias and social desirability bias. Future research could incorporate multiple data sources or objective indicators to reduce these biases. Fourth, the mediation analysis did not fully account for potential confounders. Subsequent research should incorporate additional factors, such as social support, psychological capital, and self-efficacy, into structural models to provide a more nuanced understanding of these relationships.

## Figures and Tables

**Table 1:** Factor loadings of the measurement model.

Latent Variable	Observed Variable	B	Β	S.E.	C.R.	AVE	Construct Reliability
School burnout	Difficulty adjusting to classes	1.000	0.576			1.525	0.803
	Apathy toward school life	1.028	0.646	0.050	20.411 ***		
	Identity confusion	1.175	0.646	0.052	22.769 ***		
	Career anxiety	1.211	0.781	0.054	22.462 ***		
Loneliness	Loneliness 1	1.000	0.875			1.840	0.963
	Loneliness 2	1.014	0.873	0.021	48.897 ***		
	Loneliness 3	1.158	0.896	0.023	50.783 ***		
Fear of alienation	Perceived relative deprivation	1.000	0.743			1.509	0.802
	Extrinsic motivation	1.277	0.983	0.035	36.516 ***		
	Need for belonging	0.951	0.714	0.031	31.156 ***		
Life satisfaction	Life satisfaction 1	1.000	0.776			2.598	1.454
	Life satisfaction 2	1.010	0.869	0.010	25.067 ***		

*** *p* < 0.001.

**Table 2:** Descriptive statistics and correlation analysis results.

Variable	M	SD	Skewness	Kurtosis	School Burnout	Loneliness	Fear of Alienation
School burnout	2.54	0.71	0.130	−0.162	1		
Loneliness	1.97	0.59	0.684	0.509	0.519 **	1	
Fear of alienation	2.28	0.85	0.445	−0.251	0.527 **	0.439 **	1
Life satisfaction	3.55	0.75	−0.185	−0.236	−0.426 **	−0.491 **	−0.271 **

Note. M = mean; SD = standard deviation. ** *p* < 0.01.

**Table 3:** Structural model validation.

Variable	χ^2^	*df*	NFI	TLI	CFI	RMSEA
Full multiple mediation model	492.465	47	0.958	0.946	0.962	0.073
Partial multiple mediation model	416.346	46	0.965	0.955	0.968	0.067
Model fit indices				≥0.90	≥0.90	≤0.10

**Table 4:** Structural model results.

Types	B	β	S.E.	C.R.	*p*
School burnout → Loneliness	0.461	0.620	0.021	22.297	***
School burnout → Fear of alienation	0.832	0.700	0.035	24.103	***
Loneliness → Life satisfaction	−0.486	−0.375	0.043	−11.325	***
Fear of alienation → Life satisfaction	0.096	0.119	0.032	2.973	***
School burnout → Life satisfaction	−0.390	−0.405	0.046	−8.498	***

*** *p* < 0.001.

**Table 5:** Direct, indirect, and total effects among variables.

Types		Direct Effects	Indirect Effects	Total Effects
School burnout	Loneliness	0.620		0.620 ***
	Fear of alienation	0.700		0.700 ***
	Life satisfaction	−0.405	−0.149	−0.554 ***
Loneliness	Life satisfaction	−0.375		−0.375 ***
Fear of alienation	Life satisfaction	0.119		0.119 ***

*** *p* < 0.001.

**Table 6:** Sobel test results.

Types	A	S_a_	b	S_b_	Z
School burnout → Loneliness → Life satisfaction	0.461	0.021	−0.486	0.043	−10.048 ***
School burnout → Fear of alienation → Life satisfaction	0.832	0.035	0.096	0.032	4.489 ***

Note. a and b = unstandardized path coefficients; S_a_ and S_b_ = standard errors of a and b, respectively; Z = Sobel test statistic. *** *p* < 0.001.

## Data Availability

The data are available from the corresponding author upon reasonable request.
